# Sumo and the cellular stress response

**DOI:** 10.1186/s13008-015-0010-1

**Published:** 2015-06-20

**Authors:** Jorrit M. Enserink

**Affiliations:** Institute for Microbiology, Oslo University Hospital, Sognsvannsveien 20N-0027, Oslo, Norway

**Keywords:** Sumo, Stress response, Transcription, DNA damage response, ER stress, Viral infections, Nutrient stress, SIMs

## Abstract

The ubiquitin family member Sumo has important functions in many cellular processes including DNA repair, transcription and cell division. Numerous studies have shown that Sumo is essential for maintaining cell homeostasis when the cell encounters endogenous or environmental stress, such as osmotic stress, hypoxia, heat shock, genotoxic stress, and nutrient stress. Regulation of transcription is a key component of the Sumo stress response, and multiple mechanisms have been described by which Sumo can regulate transcription. Although many individual substrates have been described that are sumoylated during the Sumo stress response, an emerging concept is modification of entire complexes or pathways by Sumo. This review focuses on the function and regulation of Sumo during the stress response.

## Introduction

Several small ubiquitin-like molecules were identified during the 1990s, including Sumo (Small ubiquitin-like modifier) [1]. Despite limited sequence similarity, Sumo is structurally related to ubiquitin with a similar protein fold. [2], although the distribution of charged residues on the surface of the Sumo molecule differs from that of ubiquitin [3]. The budding yeast *Saccharomyces cerevisiae* only expresses a single form of Sumo (encoded by the *SMT3* gene), whereas mammalian cells express four; Sumo1, −2,-3,-4.

During recent years it has become clear that Sumo has important functions in normal cell homeostasis, in large part through regulation of transcription (reviewed in Chymkowitch et al., submitted). However, Sumo is also very important for the cellular stress response, and many cellular stresses result in increased formation of Sumo conjugates. Sumo can be covalently attached to a large number of proteins to regulate their fate, localization and function. The physiological significance of many of these sumoylation events remains unknown, which is in part due to the fact that Sumo can be attached to multiple components of an entire complex, and preventing the attachment of Sumo to a single component of the complex often has little or no clear effect. Furthermore, when a given Sumo site is mutated, Sumo is sometimes attached to other sites in the same substrate with apparently little effect on the overall outcome. In contrast to the ubiquitination machinery, the sumoylation machinery only consists of very few components, raising the question how specificity is achieved and how the activity of the Sumo pathway is regulated.

The scope of this review is to provide an overview of the function of Sumo in the cellular stress response, in particular transcription, and to highlight a number of key questions that remain to be answered.

### Protein sumoylation

#### The sumoylation machinery

**Fig. 1:**
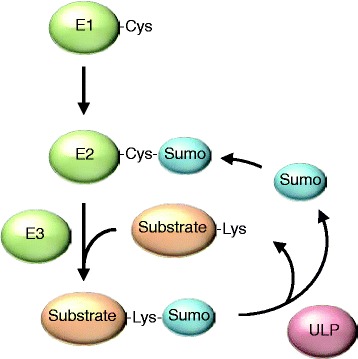
Overview of the Sumo pathway

Similar to ubiquitination, sumoylation occurs through a series of biochemical steps catalyzed by a set of well-conserved enzymes (Fig. 1 and Table 1). In the first step, Sumo, which is expressed as an inactive precursor protein, is processed by cysteine-specific Sumo proteases (ULPs in yeast, SENPs in mammals) that remove a small peptide from the C-terminus. This exposes a di-glycine motif, which is subsequently linked to the E1 enzyme, a dimer consisting of Sae1 and Sae2 (Aos1 and Uba2 in *S. cerevisiae*). This step involves the covalent attachment of Sumo to a reactive cysteine residue in Sae2 through ATP-dependent thioesterification [4]. Through thioester linkage, Sumo is then transferred to a cysteine residue of the E2 conjugating enzyme Ubc9. *In vitro*, the E2 enzyme is sufficient for conjugating Sumo to a lysine residue in the substrate, although it is believed that this process is facilitated by E3 ligases *in vivo* [4]. For instance, E3 ligases can serve as a scaffold that brings together Sumo-charged Ubc9 and the substrate, thereby promoting efficiency and specificity of the sumoylation process. Alternatively, E3 ligases can stimulate the E2 enzyme to transfer Sumo to its substrate.

**Table 1:** The sumoylation machinery in *S. cerevisiae* and mammals

Protein function	*S. cerevisiae*	*H. sapiens*
Sumo	Smt3	SUMO-1,-2,-3
E1 activating enzyme	Aos1•Uba2	Sae1•Sae2
E2 conjugating enzyme	Ubc9	UBC9
E3 ligase	Siz1, Siz2, Cst9, Mms21	PIAS1,-2,-3,-4; MZIZ1; NSE2; RanBP2; Pc2; MUL1; TOPORS; HDAC4,-7; TRAF7; FUS; RSUME
Sumo protease	Ulp1, Ulp2	SENP1,-2,-3,-4,-5,-6,-7; DESI1,-2; USPL1

An important aspect of protein sumoylation is that it is a dynamic and reversible process. Sumoylated proteins can be desumoylated by the same proteases that convert the inactive Sumo precursor to its reactive form (ULPs/SENPs). These enzymes have important functions in spatial regulation of Sumo turnover [5], which is crucial for many cellular processes including chromosome cohesion, mitosis and transcription [6–8]. In *S. cerevisiae*, the activity of Ulp1 and Ulp2 towards sumoylated proteins is in large part dependent upon their localization; Ulp1 activity in particular appears to be highly localized at nuclear pore complexes, whereas Ulp2 may be more active towards proteins located in the nucleoplasm [9, 10]. However, how the enzymatic activity of Sumo proteases is regulated is currently not well understood (also see below).

#### Consensus motifs for sumoylation

Sumoylation of substrates preferentially occurs on a lysine residue in the canonical Sumo consensus motif ΨKx(D/E), in which Ψ is a large hydrophobic residue and x is any amino acid followed by an acidic residue [11]. The hydrophobic and acidic residues promote stability of the interaction between the substrate and the E2 enzyme [12–14]. Several variations on this sumoylation motif have been identified, including so-called negatively charged amino acid-dependent Sumo motifs (NDSMs) and phosphorylation-dependent Sumo motifs (PDSMs). PDSMs are basically extended versions of the canonical Sumo motif (ψKx(D/E)xxSP), and phosphorylation of this motif by proline-directed kinases generally increases sumoylation efficiency [15]. Phosphorylated PDSMs and NDSMs likely promote sumoylation efficiency by increasing the stability of the interaction between Ubc9 and the substrate, because the negatively charged phosphate (in case of PDSM) or the negatively charged amino acids (NDSM) interact with basic residues on the surface of Ubc9 [15, 16]. It is important to note that sumoylation can also occur on lysines that do not conform to known Sumo consensus motifs [17–19], such as the well-studied K164 Sumo site in PCNA [20], and data from high-throughput studies indicate that non-consensus sumoylation may be a relatively common event [19, 21]. How these sites are recognized by the sumoylation machinery remains to be determined.

#### Sumo chains

While conjugated Sumo is probably best studied in its a monomeric form, Sumo can also form oligomeric chain structures. In *S. cerevisiae*, these are primarily formed through K15 linkage (K11 in mammalian Sumo-2/3), which is part of the canonical Sumo consensus motif, and requires the E1 enzyme and Ubc9 [22, 23]. Sumo chains are best characterized in their role as an indirect protein degradation signal; they can recruit conserved E3 ubiquitin ligases known as Sumo targeted ubiquitin ligases (STUbLs), which subsequently ubiquitinate the polysumoylated protein to target it for proteasomal degradation [24]. Some examples of STUbL targets are PML, c-Myc, the viral Tax protein, the *Drosophila* transcriptional repressor Hairy, the *S. cerevisiae* basal transcription factor Mot1, and the *A. thaliana* transcriptional repressor CDF2 [25–29]. Apart from its function in protein degradation, the physiological significance of Sumo chains remains poorly understood. A global study of Sumo chain function in *S. cerevisiae* indicates that Sumo chains are involved in regulation of transcription and ordering of chromatin structure [30], although the molecular mechanisms have not been characterized in detail.

#### Sumo interaction motifs

Sumoylation of proteins adds a bulky moiety that can affect the interaction with other proteins. For instance, sumoylation of PCNA on K127 prevents the interaction between PCNA and the chromatin cohesion protein Eco1 [31]. In another example, the covalent conjugation of SUMO-1 to lysine 341 of the base excision repair enzyme thymine DNA glycosylase (TDG) blocks the interaction with the histone acetyltransferase CBP/p300 [32]. However, most studies have focused on the function of Sumo in promoting protein-protein interactions. Here, Sumo can provide an interaction surface for specific binding partners, which typically interact with Sumo through a Sumo interaction motif (SIM). The first reported SIM was identified through a two-hybrid screen that used the p53 family member p73α as bait [33]. It was found that p73α became sumoylated in yeast, and this sumoylated form of p73α subsequently interacted with various mammalian interaction partners. By comparing common motifs in these interaction partners, it was found that they share a common ΦΦxSxS[D/E][D/E][D/E], where Φ is a hydrophobic amino acid [33]. Because this motif is centered on the two serines it became known as the ‘SxS’ motif. However, it was later found that these two serines are not critical for Sumo binding, and that the two hydrophobic amino acids play a much more important role; in fact, NMR spectroscopic characterization of the interaction between mammalian SUMO-1 and peptides derived from known Sumo binding proteins identified a hydrophobic core with the consensus [V/I]x[V/I][V/I] [34]. Subsequent studies confirmed the importance of this hydrophobic core, and structural studies have shown that in complex with Sumo the hydrophobic side chains of the SIM interact with a hydrophobic pocket on the SUMO surface [35, 36]. This canonical SIM is often flanked by acidic amino acids [37, 38], not unlike the originally reported SxS motif [33]. In some cases, phosphorylated serine residues fulfill the function of these acidic amino acids. These phospho-SIMs have been identified in PML, EXO9 and in the PIAS proteins, which are phosphorylated by the constitutively active casein kinase 2 (CK2) [39]. The phosphorylated residues in the SIM interact with a lysine residue on the Sumo surface to stabilize the SIM-Sumo interaction [39]. In addition to providing the cell with a mechanism for temporal and spatial control of protein sumoylation, phospho-SIMs may add specificity to selection of appropriate Sumo substrates.

Interestingly, the Sumo pathway often targets multiple components of protein complexes and pathways [40–42]. This phenomenon of protein group sumoylation was first described for yeast septins and was more recently also observed in the DNA checkpoint/repair pathways. The exact physiological significance of protein group sumoylation is presently not clear, although it has been proposed that multiple Sumo-SIM interactions serve as a ‘Sumo glue’ to stabilize the integrity of the complex [42]. Perhaps protein group sumoylation, in which multiple relatively weak Sumo-SIM interactions cooperate to provide increased complex stability, is important for buffering the system. More specifically, it is likely that significant Sumo noise exists in the cell, because sumoylation is a common event (up to 10% of all mammalian proteins may be Sumo targets [43]); potential SIMs are ubiquitous (4892 instances of the [V/I]x[V/I][V/I] motif can be found in the *S. cerevisiae* proteome, totaling 2919 proteins); and under normal growth conditions a large pool of free Sumo exists in the cell, which could compete with sumoylated proteins for binding to SIMs. To overcome this noise, protein group sumoylation may be a strategy of the cell to increase the signal-to-noise ratio of Sumo-SIM interactions to generate a consistent output.

### The Sumo stress response

Early studies with mammalian and yeast cells found that Sumo is important for the cellular response to stress, such as heat shock, DNA damage, oxidative stress and ethanol stress [44, 45]. Although Sumo is crucial for the stress response in plants, here I will mainly focus on studies conducted in yeast and vertebrates, referring the reader to recent reviews on Sumo in the plant stress response [46–48].

#### DNA damage response

**Fig. 2:**
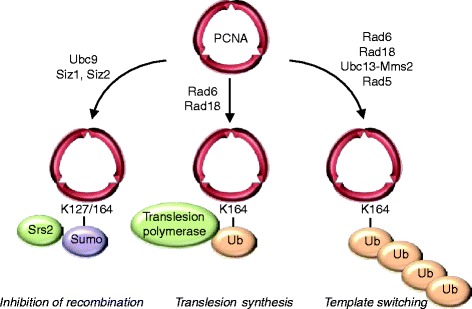
Regulation of PCNA by ubiquitin and Sumo. When cells are treated with high doses of MMS, PCNA becomes Sumo-modified primarily on K164 and to a lesser extent K127, resulting in recruitment of HR inhibitor Srs2. At lower levels of DNA damage PCNA is ubiquitinated mainly on K164 (and to a lesser degree also on other sites [113, 51]). This promotes lesion bypass in case of monoubiquitination, whereas polyubiquitinated PCNA induces template switching and error-free DNA repair

Sumo has been studied extensively in the context of DNA damage, and except for a few specific examples I will not discuss this in great detail here and instead refer the reader to recent reviews [49, 50]. One of the best known targets of Sumo in the DNA damage response is PCNA[20]. PCNA is a trimeric complex that functions as a sliding clamp and processivity factor for DNA polymerases. In response to endogenous and exogenous DNA damage PCNA can be modified by ubiquitin and Sumo [20, 51] (Fig. 2). Sumoylation occurs preferentially on the evolutionarily conserved K164, and to a lesser extent on the yeast-specific residue K127 [20]. Sumoylated PCNA recruits the helicase Srs2, which has a SIM in its extreme C-terminus. Srs2 is an inhibitor of homologous recombination (HR) [52, 50, 53], and recruitment of Srs2 is believed to suppress undesirable recombination events during chromosome replication [54]. Srs2 itself is also a target of Sumo [55], and sumoylation of Srs2 appears to interfere with binding of Srs2 to sumoylated PCNA [56], although the exact physiological consequences for the DNA replication and repair process remain unclear.

In addition to PCNA and Srs2, many other DNA damage response proteins are sumoylated in response to DNA damage [50, 57]. A recurrent theme appears to be protein group sumoylation [40, 42]. For instance, exposure of single stranded DNA (ssDNA) induces sumoylation of several proteins involved in HR, which is thought to promote DNA double strand break repair. For at least some of these proteins, simply localizing to the chromatin compartment is sufficient for sumoylation to occur, probably because it results in their colocalization with the chromatin-bound E3 ligase Siz2. Why Siz2 specifically sumoylates HR proteins and not any other chromatin-bound protein remains to be understood.

#### Viral infections

Infections with pathogens like viruses trigger a major stress response. Interferon (IFN) plays a central role in the response to viral infections. At the cellular level, IFN has a number of effects, most notably an increase in the number and size of so-called promyelocytic leukemia protein-nuclear bodies (PML-NBs). PML-NBs are dynamic nuclear substructures that consist of a very large number of proteins centered around the PML protein, which is essential for organizing these proteins into PML-NBs [58, 59]. Although the PML protein has been extensively studied in the context of acute promyelocytic leukemia, in which it is fused to the retinoic acid receptor to drive cancer cell proliferation and survival, in healthy cells it fulfills many functions essential for normal cell homeostasis. For instance, PML-NBs have been implicated in transcription, mRNA transport, the DNA damage response, telomere maintenance, the cellular stress response, apoptosis, stem cell maintenance and senescence [60, 61, 59]. PML-NBs also serve an important function in the antiviral response [60]. For instance, PML−/− mice exhibit increased viral load after infection with lymphocytic choriomeningitis virus (LCMV) and vesicular stomatitis virus (VSV) [62]. One way PML-NBs inhibit VSV infections is by recruiting and activating p53 to induce apoptosis, thereby killing the infected cell to protect the organism [60].

**Fig. 3:**
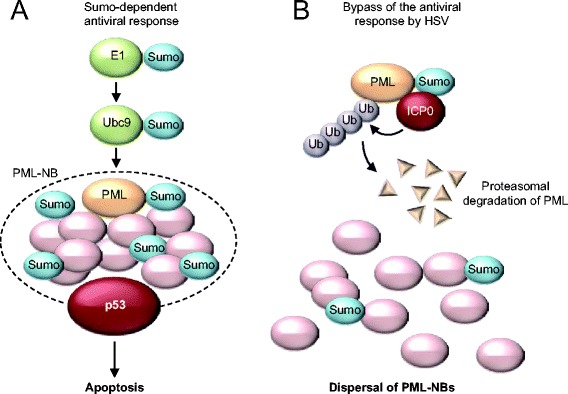
Viruses can target the Sumo pathway to disrupt PML-NBs. **a**, Several components of PML-NBs are targets of Sumo, and multiple Sumo-SIM interactions may promote complex stability. **b**, The HSV viral protein ICP0 can function as a STUBL to degrade sumoylated PML, thereby disrupting structural integrity of PML-NBs

Sumo is a key regulator of PML function [59]. PML directly binds Ubc9, resulting in sumoylation on at least three lysines, K65, K160 and K490. Interestingly, PML has Sumo E3 ligase activity [63], which may mediate sumoylation of many components of PML-NBs [59]. It is believed that protein group sumoylation of these factors promotes the assembly of PML-NBs through multiple Sumo-SIM interactions [59] (Fig. 3a). Indeed, sumoylation of PML is essential for formation of PML-NBs, and a non-sumoylatable PML mutant fails to recruit key components of PML-NBs, including the transcriptional regulators SP100 and DAXX [59].

The importance of Sumo in the antiviral stress response is highlighted by the fact that sumoylation of PML increases upon infection with poliovirus, leading to recruitment of p53 and induction of apoptosis [64]. Not surprisingly, viruses have developed several mechanisms that disrupt PML-NBs, some of which target the sumoylation machinery. For instance, the chicken embryo lethal orphan (CELO) virus, which is an avian adenovirus, expresses a protein called Gam-1. Gam-1 blocks the sumoylation pathway by inhibiting formation of the E1-SUMO thioester-intermediate and by promoting proteasomal degradation of E1 and E2 proteins [65, 66].

Two other examples of viral proteins that target the Sumo pathway to disperse PML-NBs are the herpes simplex virus ICP0 protein and the human cytomegalovirus IE1 protein. Both these proteins interfere with sumoylation of PML, resulting in disassembly of PML-NBs [67]. ICP0 is believed to function as a STUbL that specifically binds Sumo-modified proteins including PML, resulting in their ubiquitination to target them for proteasomal degradation (Fig. 3b). Many other viruses have developed mechanisms that thwart the PML-NB antiviral defense system [68, 69]. Taken together, these studies show that Sumo plays an important role in the cellular antiviral stress response.

#### Endoplasmic reticulum (ER) stress response

Anomalies in ER function can result in the accumulation of misfolded proteins, which is often referred to as ER stress. ER stress results in activation of the unfolded protein response (UPR), which aims to re-establish cellular homeostasis by reducing the amount of unfolded proteins. Notably, while the UPR promotes cell viability at low doses of ER stress, it can also induce apoptosis when ER stress is not mitigated.

**Fig. 4:**
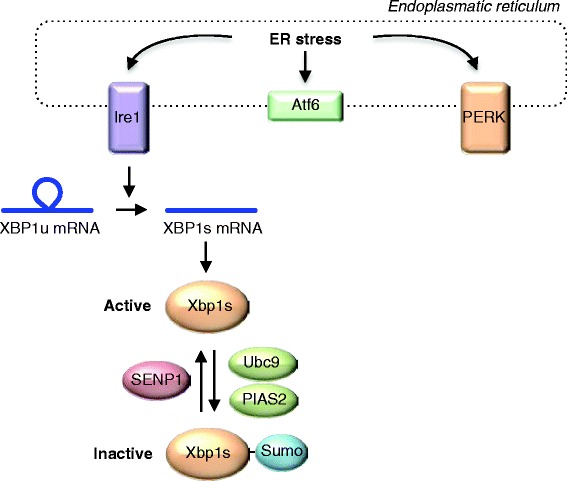
Sumo and the ER stress response. Unfolded proteins trigger the ER stress response, inducing processing of XBP mRNA by Ire1, ultimately yielding active XBP1s. Sumoylation of XBP1s and the physical interaction with Ubc9 inhibit its transcriptional activity

As illustrated in Figure 4, the UPR consists of three branches, i.e. inositol-requiring protein 1 (IRE1), protein kinase RNA-like ER kinase (PERK) and activating transcription factor 6 (ATF6) [70]. IRE1 contains both a kinase and an endoribonuclease domain. ER stress induces dimerization and autophosphorylation of IRE1, leading to activation of the cytosolic RNase domain. The RNase domain subsequently excises a small intron from the mRNA encoding the transcription factor X box-binding protein 1 (XBP1). This alters the reading frame of the mRNA, resulting in translation of the active transcription factor, known as ‘spliced XBP1’ (XBP1s). XBP1s then activates its target genes, which have important functions in the ER-associated protein degradation (ERAD) pathway, ER protein import, protein folding, and lipid synthesis [70].

Interestingly, the Sumo pathway has multiple effects on the UPR. For instance, in mammalian cells XBP1s is sumoylated by PIAS2 on two lysines in the C-terminal transactivation domain, which inhibits the transcriptional activity of XBP1s [71]. Upon ER stress, the Sumo protease SENP1 desumoylates XBP1s to promote its transcriptional activity [72]. This effect of Sumo on XBP1 appears to be conserved in *C. elegans* [73]. Furthermore, mRNA encoding Sumo is degraded upon ER stress in Drosophila [74], and although the physiological consequences remain unknown, it is tempting to speculate that depletion of Sumo further boosts the activity of XBP1s. Finally, binding of mammalian Ubc9 to the leucine zipper motif of XBP1s increases XBP1s stability to enhance its transcriptional output, although this effect seems to be independent of the catalytic activity of Ubc9 [75]. Together, these findings show that the Ubc9-Sumo pathway has a negative effect on the ER stress response.

#### Regulation of transcription by Sumo

We have recently reviewed the diverse functions of Sumo in transcriptional regulation during normal growth conditions (Chymkowitch et al., submitted), and here I will mainly focus on regulation of transcription during cell stress.

**Fig. 5:**
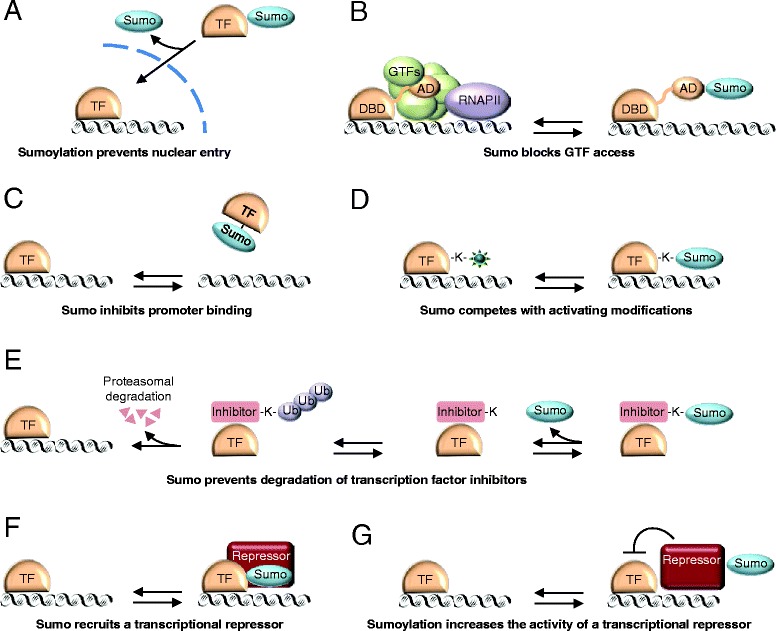
Multiple mechanisms of transcriptional regulation by Sumo. **a**, Sumoylation prevents nuclear entry. **b**, Sumo prevents recruitment of general transcription factors (GTFs). **c**, Sumo inhibits promoter binding of the transcription factor. **d**, Sumo competes with other modifications that activate transcription. **e**, Sumo prevents degradation of an inhibitor of a transcription factor. **f**, Sumo recruits a transcriptional repressor that silences the local chromatin environment. **g**, Sumoylation increases the activity of a transcriptional repressor to inhibit transcription

Sumo is best known for its inhibitory function in transcription [76]. Several mechanisms have been described by which Sumo can inhibit transcription (Fig. 5). For instance, as has been described for the transcription factor Atf7 [77], Sumo can inhibit nuclear entry of transcription factors (Fig. 5a); it can prevent recruitment of general transcription factors (Fig. 5b); or it can block binding of transcription factors to specific sequences in the promoter (Fig. 5c). The sumoylation machinery can also inhibit transcription by competing for lysines that are targets for other modifications associated with transcriptional activation, such as acetylation, methylation or ubiquitination (Fig. 5d). This has been reported for STAT5A, where inhibitory sumoylation competes with an activating acetyl modification of K696 [78]. In a related mechanism, Sumo can prevent ubiquitin-mediated degradation of transcriptional inhibitors (Fig. 5e), as was described for IκBα [79]. IκBα is an inhibitor of the transcription factor NFκB, and IκBα can be ubiquinated on K21, which results in its proteasomal degradation. This relieves inhibition of NFκB, which subsequently activates transcription. However, K21 of IκBα is also a target for sumoylation, and Sumo-modified IκBα prevents ubiquitin-mediated degradation of IκBα, thereby preventing activation of NFκB [79]. Sumoylation of transcription factors can also result in recruitment of transcriptional repressors (Fig. 5f). An example of this mechanism is sumoylation of the transcription factor Elk-1, which results in recruitment of HDAC-2, which silences chromatin by deacetylating histones [80]. Transcriptional repressors can themselves be activated by sumoylation to create a repressive chromatin environment (Fig. 5g); for example, sumoylation of HDAC1 promotes transcriptional repression *in vivo* [81].

Sumo-mediated inhibition of transcription is likely to be an important aspect of the cellular stress response. For instance, various stresses including heat shock, ethanol treatment and osmotic stress induce PIAS-mediated sumoylation of c-Myb, which results in inhibition of its transcriptional activity [82]. Since c-Myb is a major regulator of cell proliferation, unfavorable conditions may induce sumoylation of c-Myb to switch off the transcriptional programs required for cell proliferation.

Sumo can also activate transcription during cell stress. For instance, sumoylation of the heat shock transcription factors HSF1 and HSF2 increases their DNA binding activity, resulting in increased expression of heat shock proteins which provide protection against protein-damaging stress [83, 84]. Another example is the activation of NFκB in response to genotoxic stress. Genotoxic stress induces sumoylation and nuclear localization of NEMO, an activator of the IκB kinase IKK. This NEMO-induced IKK activation leads to phosphorylation of the NFκB inhibitor IκB, resulting in proteasomal degradation of IκB and activation of NFκB to transcribe pro-survival genes [85, 86].

Together, these studies illustrate the complexity of Sumo’s function in regulating transcription during the cellular stress response, and it is largely unclear how specificity is achieved in this process (also see below).

#### Sumo and the nutrient response

**Fig. 6:**
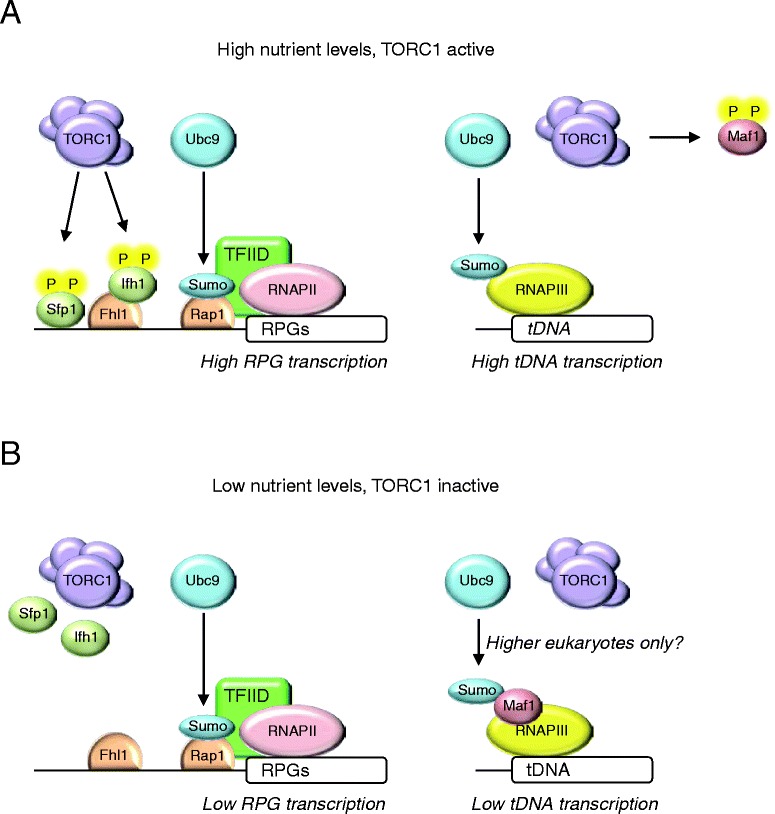
Regulation of pro-growth genes by Sumo. **a**, in the presence of sufficient nutrients, TORC1 and Ubc9 in transcription of pro-growth genes like RPGs and *tRNA* genes. TORC1 increases phosphorylation of Sfp1 and Ifh1, leading to their recruitment to PRG promoters. Ubc9 sumoylates Rap1, which enhances recruitment of TFIID to RPGs. Ubc9 also sumoylates RNAPIII components, which most likely is required for efficient *tRNA* transcription. TORC1 increases phosphorylation of Maf1, resulting in its nuclear exclusion. **b**, During nutrient stress TORC1 is inactive, leading to dephosphorylation of Sfp1 and Ifh1, which are then released from RPG promoters. Maf1 also becomes dephosphorylated, resulting in its nuclear entry where it binds and inhibits RNAPIII. At least in mammals Maf1 is also sumoylated, which contributes to its repressive effect on RNAPIII. Whether this also occurs in yeast is unknown

One major source of stress is a limitation in nutrient availability. Maintaining cellular homeostasis in the face of changes in nutrient supply is essential for the growth and development of all organisms, from unicellular microorganisms to higher eukaryotes. Cellular mechanisms have evolved that sense environmental alterations and evoke responses that act to preserve homeostasis. When a cell detects a reduction in its nutrient supply, it activates signal transduction pathways that elicit integrated responses that alter cell metabolism by reducing biosynthesis and increasing catabolism, and that mobilize utilization of alternative nutrient sources. One key element of the response to nutrient starvation is rewiring of gene expression programs. Target of rapamycin complex 1 (TORC1), a large protein complex that contains the Tor kinase, play a central role in the cellular response to nutrient status [87–89]. TORC1 activity strongly depends on nutrient availability, in particular nitrogen. Under nitrogen-rich conditions, TORC1 promotes growth and proliferation-related processes, like protein synthesis, ribosome biogenesis, and tRNA synthesis, while inhibiting catabolic processes, like autophagy [87–89]. Conversely, inhibition of TORC1 activity by nutrient depletion (or addition of the TORC1-specific inhibitor rapamycin) results in a metabolic switch from anabolism to catabolism [87–89]. This involves many cellular processes, including rewiring of transcriptional programs [87, 90]. An important set of genes whose expression depends upon TORC1 includes genes involved in regulation of translation, such as the RNAPII-transcribed ribosomal protein genes (RPGs) and the RNAPIII-transcribed *tRNA* genes. In *S. cerevisiae*, TORC1 activates RPG transcription by promoting phosphorylation of the transcription factors Sfp1 and Ifh1, leading to their recruitment to PRG promoters where they activate transcription together with several other transcription factors, including Rap1 and Fhl1 (Fig. 6a) [91–94]. In addition, TORC1 regulates transcription of RNAPIII-dependent genes by phosphorylating the transcriptional repressor Maf1, which results in nuclear exclusion of Maf1 (Fig. 6a) [88]. Nutrient stress results in inactivation of TORC1, leading to dephosphorylation of Sfp1, Ifh1 and Maf1. Subsequently, dephosphorylated Sfp1 and Ifh1 leave RPG promoters, resulting in loss of RPG transcription, whereas dephosphorylation of Maf1 causes it to enter the nucleus where it binds and inhibits RNAPIII to block transcription of *tRNA* genes (Fig. 6b) [88, 89]. This ultimately results in downregulation of the translational capacity of the cell until nutrient conditions improve [89].

We recently found that nutrient stress profoundly affects cellular sumoylation patterns in yeast [95]. Nutrient stress particularly inhibits sumoylation of several transcription factors, especially components of the RNAPIII polymerase. Preventing sumoylation of RNAPIII results in strongly reduced *tRNA* expression levels [95]. Although the molecular mechanism remains to be solved, these findings indicate that sumoylation of RNAPIII is required for its activity, and that nutrient stress inhibits *tRNA* transcription by preventing sumoylation of RNAPIII [95]. This may be conserved in human cells, because several RNAPIII components have been identified as Sumo targets in high-throughput proteomic studies, although the functional consequences of these modifications have not been studied [96–99].

Interestingly, in human cells Sumo is also associated with inhibition of RNAPIII-dependent transcription upon nutrient stress. Specifically, downregulation of RNAPIII during nutrient starvation requires sumoylation of Maf1, and cells expressing a non-sumoylatable mutant of Maf1 do not fully repress *tRNA* transcription during starvation [100]. These studies indicate that Sumo may differentially regulate RNAPIII activity, although more studies are required to fully understand how Sumo regulates RNAPIII.

Another yeast transcription factor that is regulated by Sumo is Rap1 [95, 101]. Rap1 becomes sumoylated on multiple sites, and Sumo increases the transcriptional activity of Rap1 at RPGs by promoting the recruitment of TFIID [95]. Although the exact molecular mechanism of TFIID recruitment to sumoylated Rap1 remains to be revealed, most components of the TFIID complex contain one or several SIMs, indicating that Sumo-SIM interactions may be important.

Paradoxically, whereas nutrient stress results in decreased RPG transcription, Rap1 sumoylation is increased under these conditions [95]. This is potentially the result of a homeostatic feedback loop, where the cell senses a decrease in its translational capacity and responds by attempting to restore RPG transcription by sumoylating Rap1. Indeed, Rap1 sumoylation is essential for maintaining a basal rate of RPG transcription under nutrient stress, and preventing Rap1 sumoylation during nutrient stress completely abolishes RPG transcription, causing a strong decrease in cell viability [95].

Regulation of RPGs by Sumo is conserved in mammalian cells, although the critical target is not the human homolog of Rap1 but rather the transcription factor scaffold attachment factor b1 (SAFB1). Together, these studies show that Sumo plays an important role in promoting cell growth and proliferation and that it is strongly affected by the cell’s nutrient status.

#### How is the Sumo pathway activated by stress, and how is specificity achieved?

It is still not very clear how specificity is achieved in the Sumo pathway. A very large number of proteins can be sumoylated, and an even larger number of proteins contains potential SIMs. Yet, specific stresses induce sumoylation of specific sets of proteins, and these sets of proteins may be different between various organisms. For instance, in yeast, DNA damage mainly induces sumoylation of DNA damage response proteins, such as proteins involved in HR [42], whereas in human cells the Sumo network primarily consists of chromatin modifiers and transcription factors [102].

In yeast, specificity towards HR proteins has been suggested to involve colocalization of Siz2 and HR proteins at ssDNA, and artificial targeting of HR proteins to DNA is sufficient to trigger their sumoylation [42]. Exactly how Ubc9 and Siz2 are regulated in this context remains unclear. It is possible that phosphorylation helps coordinate the Sumo pathway. In yeast, DNA damage checkpoint kinases, such as Tel1 (yeast ATM), Mec1 (yeast ATR), Chk1 and Rad53 (yeast Chk2) phosphorylate many proteins involved in the DNA damage response, including HR proteins [103]. Some of these phosphorylations may occur in PDSMs to promote subsequent sumoylation. Phosphorylation-mediated coordination of protein sumoylation could be a more general model for the Sumo stress response, since cellular stress activates several stress-induced kinases, such as p38 and JNK in mammals and Slt2 and Hog1 in yeast.

Locally, the activity of components of the sumoylation machinery may be regulated by post-translational modifications. In yeast, the E1 enzymes Uba2 and Aos1 have been found to be phosphorylated, although the functional consequences have not been explored [104–106].

The E2 conjugase Ubc9 can be activated by phosphorylation. In mammalian cells, Akt phosphorylates Ubc9 on T35, which promotes thioester bond formation between Ubc9 and Sumo to increase Ubc9 activity [107]. This also promotes sumoylation of Ubc9 itself, which is believed to be important for substrate selection [108]. In addition to Ubc9, Akt can also phosphorylate Sumo-1 (on T76), which promotes the stability of Sumo-1. Thus, phosphorylation both activates Ubc9-Sumo and increases specificity of the sumoylation pathway. Cdk1 can also activate Ubc9 [109], although it phosphorylates Ubc9 on a different residues than Akt, i.e. S71, which falls within a full cyclin B-Cdk1 consensus site [110]. In contrast to phosphorylation by Akt, Cdk1-dependent phosphorylation does not result in increased autosumoylation of Ubc9 [109]. For both Akt- and Cdk1-dependent phosphorylation, the exact molecular mechanism by which phosphorylation activates Ubc9 remains to be revealed.

Other components of the sumoylation machinery are also kinase targets. For instance, the E3 ligase PIAS1 is phosphorylated by IKK on S90, which results in its localization to NF-κB-bound promoters where it inhibits transcription [111]. Furthermore, in yeast the desumoylating enzymes Ulp1 and Ulp2 have been found to be extensively phosphorylated, although the physiological relevance is unknown [106, 104, 103].

Finally, it worth mentioning that it was recently reported that in yeast the Sumo stress response critically depends upon active transcription, but does not require translation [112]. The authors observed that the osmotic shock-induced Sumo stress response does not appear to be directly linked to the stress itself, but rather represents a synchronized wave of sumoylation that occurs as a consequence of large-scale, coordinated changes in the transcriptional program in response to environmental stress [112]. This is an interesting observation, but it is presently difficult to understand how such transcriptional changes would result in sumoylation of specific protein complexes; for instance, why do osmotic shock-induced transcriptional changes not result in sumoylation of HR proteins, or other Sumo targets like septins [41]? More detailed follow-up studies are required to understand how active transcription is linked to protein group sumoylation.

## Conclusions

Together, these studies show that Sumo plays an important role in maintaining cell homeostasis. Under optimal conditions, Sumo promotes cell growth and proliferation by activating pro-growth genes, whereas during cell stress Sumo contributes to activation of pro-survival pathways. Nonetheless, a lot remains to be learned about the Sumo stress response and regulation of the sumoylation machinery by phosphorylation and other post-translational modifications. Another major question that remains to be answered is how specificity is achieved.
